# THRU–REFLECT Method for Scattering Parameter Extraction from Back-to-Back Measurements of Waveguide Components

**DOI:** 10.3390/s25072277

**Published:** 2025-04-03

**Authors:** Songyuan Xu, Jiwon Heo, Chan-Soo Lee, Ki-Hong Kim, Bierng-Chearl Ahn

**Affiliations:** School of Electric and Computer Engineering, Chungbuk National University, Cheongju 28644, Republic of Korea; 2021197002@chungbuk.ac.kr (S.X.); heo1234@chungbuk.ac.kr (J.H.); direct@chungbuk.ac.kr (C.-S.L.)

**Keywords:** *S*-parameter extraction, back-to-back measurement, waveguide components

## Abstract

The design of new waveguide components is often verified by back-to-back measurements of two identically fabricated units without extracting the characteristics of a single device. This paper presents a simple method of extracting the scattering parameters of waveguide components from back-to-back measurements. The proposed method requires only three waveguide mating connections: one for reflection measurement with an offset SHORT and two for transmission measurement with a THRU configuration. A singular condition in the *S*-parameter extraction equations is derived, and the optimum length of an offset SHORT standard or a reflecting load is determined based on the singularity condition. The numerical simulation of a broadband coax-to-waveguide transition is employed to show the workings of the proposed method.

## 1. Introduction

The design of new two-port waveguide components is often experimentally verified using back-to-back measurements of two identically fabricated units. The scattering parameters of a two-port fixture or device can be obtained from reflection measurements using three standard loads: SHORT, OPEN, and MATCH [1,2]. When standard loads are not readily available, it is convenient to use two symmetrical or identical devices in a back-to-back configuration. There is a large number of studies that employ back-to-back measurements for the experimental verification of microwave devices. The back-to-back method is very popular for the experimental verification of transitions between different types of transmission lines, such as coaxial-to-waveguide transition and waveguide-to-printed circuit transition [3,4,5,6,7,8,9,10,11,12,13,14,15]. It has been used for other waveguide devices as well [16,17,18]. In the literature, authors often present their results using back-to-back measurements of two symmetric modules, leaving out the task of extracting the measured scattering parameters of a single unit of the designed device. In device characterization using transmission line transitions, it is necessary to extract the scattering parameters of a single transition from back-to-back measurements or from other types of measurements.

In order to extract the scattering parameters of a single transition or device from back-to-back measurements, one can use various calibration or unterminating methods, including the classic TRL (THRU–REFLECT–LINE) method and its variants [19,20,21,22,23,24,25]. They can be used for the characterization of two non-identical back-to-back connected devices, as well as two identical ones. These methods, however, are overkill since there are only three unknowns, *S*_11_, *S*_22_, and *S*_21_, which can be determined by two measurements: a one-port measurement and a two-port measurement that are independent of each other. One may use time–domain-based methods such as the 1x-REFLECT method and the 2x-THRU method for asymmetrical or symmetrical devices in a back-to-back configuration [26,27,28]. They, however, require a vector network analyzer with a large bandwidth along with time–domain measurement software functionality. The device size needs to be large enough for the time–domain separation of the reflections caused by a single device, which is a factor that makes the 2x-THRU method unattractive for millimeter- and sub-millimeter-wave frequencies, as the device size is often too small for time–domain gating [26].

For symmetrical devices measured in a back-to-back configuration, there are two major frequency–domain approaches to calibrating or characterizing a single device: the LINE–REFLECT [29] method and the LINE–LINE method [30]. The LINE measurement means obtaining the two-port scattering parameters of two identical devices with a section of a transmission line with a known transmission coefficient inserted between the two devices. A back-to-back configuration without a line between the two devices is called THRU. The REFLECT procedure refers to a one-port measurement with a reflecting load with a known reflection coefficient, Γ, connected to the port to mate the two devices. A general REFLECT standard includes a matched load (Γ = 0) [31,32].

In waveguide measurements, misalignment in waveguide-to-waveguide connection is a significant contributor to measurement error, especially above millimeter-wave frequencies. Therefore, a procedure that requires fewer waveguide connections is preferable. Here, a connection means one contact between two waveguide transverse planes or flanges. The THRU–REFLECT configuration requires only three flange-mating connections, two for the THRU measurement and one for the REFLECT measurement, while the LINE–LINE method requires four connections. In the THRU–REFLECT method, the REFLECT measurement provides one equation for *S*-parameter extraction. The THRU measurement yields the remaining two equations necessary for complete *S*-parameter extraction.

When a two-port device has coaxial and waveguide ports, the scattering parameters of a single unit can be obtained from back-to-back measurements by adding an additional measurement of the reflection coefficient with an offset SHORT connected to the waveguide port [33,34]. The offset SHORT load is the easiest standard to implement in a hollow metal waveguide. It is a perfect standard with a well-defined shorting position with no parasitic effects.

It is the aim of this paper to present a simple method for extracting the scattering parameters of a waveguide component from back-to-back measurements using the THRU–REFLECT method. The method requires only one reflection measurement with an offset SHORT before or after the back-to-back measurement. Although the demonstration of the measurement is shown for a rectangular waveguide device, the proposed method can be applied to the characterization of other types of waveguides, such as circular waveguides, two-wire transmission lines, and printed lines (e.g., coplanar waveguides).

Many sensors include one or more transitions or interfaces, whose characteristics need to be characterized. The concept proposed in this paper can also be applied to the characterization of sensor interfaces as far as they can be formulated in terms of the scattering parameters.

The proposed method, albeit simple, has never been formally applied to back-to-back measurements in the open literature to the best knowledge of the authors. First, we present the equations for scattering parameter extraction from the back-to-back measurements of two symmetrical devices. Then, singularities in the *S*-parameter extraction equation are investigated, from which a formula for the optimum length of an offset SHORT or a reflecting load is obtained. Next, an example of the application of the proposed method is presented using a transition from a coaxial line to a reduced-height rectangular waveguide. Finally, the conclusions of the paper are drawn.

## 2. Theory of Scattering Parameter Extraction

**Figure 1:**
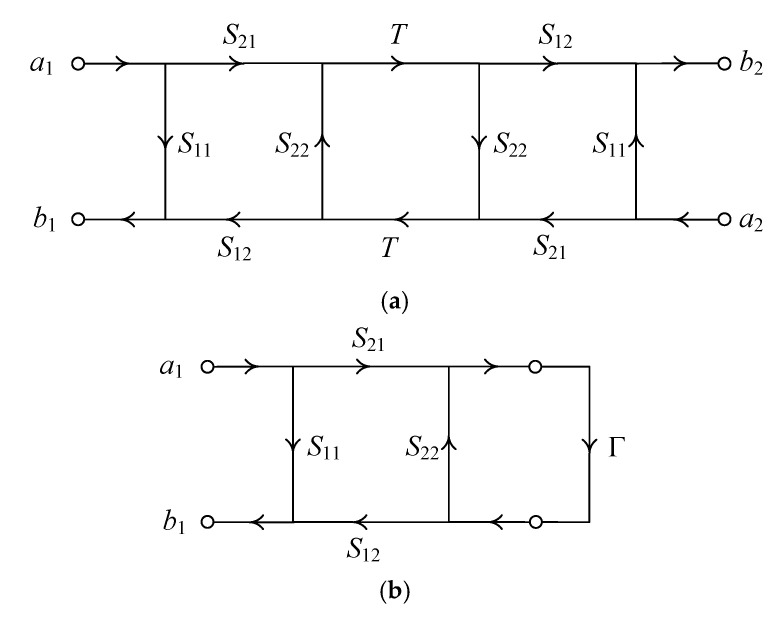
Signal flow graph for the THRU measurement (**a**) and for the REFLECT measurement (**b**).

Figure 1a shows a signal flow graph representation of a THRU measurement of two symmetric devices in a back-to-back configuration, where *a*_1_ and *b*_1_ are the incident and reflected waves at Port 1, respectively, while *a*_2_ and *b*_2_ are those at Port 2, respectively. The scattering parameters of the device are denoted as *S*_11_, *S*_21_, *S*_22_, and *S*_12_, where *S*_11_ and *S*_21_ are the reflection coefficient at Port 1 and the transmission coefficient from Port 1 to Port 2 with Port 2 terminated with a matched load, respectively, and *S*_22_ and *S*_12_ are the reflection coefficient at Port 2 and the transmission coefficient from Port 2 to Port 1 with Port 1 terminated with a matched load, respectively. The transmission coefficient of the THRU standard is represented by *T*, which is one in an ideal case. The THRU standard has zero reflection. Figure 1b shows a signal flow graph for the REFLECT measurement of a device terminated with a load with reflection coefficient, Γ. The calibration load in the REFLECT measurement can, in general, arbitrarily include a matched termination (Γ = 0).

The unknown parameters to be determined from the measurements are *S*_11_, *S*_22_, and *S*_21_*S*_12_. For reciprocal devices, *S*_21_ (=*S*_12_) can be derived from *S*_21_*S*_12_ using rough information on the phase of *S*_21_, which can be estimated from the physical dimension of the device. Individual values of *S*_21_ and *S*_12_ are usually not necessary since they are used in the product form *S*_21_*S*_12_ in the de-embedding work.

From the THRU measurement shown in Figure 1a, we obtain the following measured scattering parameters from the signal flow graph analysis [35]:

(1) $$M_{11}={\left.\frac{b_{1}}{a_{1}}\right|}_{a_{2}=0}=S_{11}+\frac{S_{21}S_{12}T^{2}S_{22}}{1-S_{22}^{2}T^{2}}\;$$

(2) $$M_{21}={\left.\frac{b_{2}}{a_{1}}\right|}_{a_{2}=0}=\frac{S_{21}S_{12}T}{1-S_{22}^{2}T^{2}}$$

where *M*_11_ is the measured reflection coefficient at Port 1 with Port 2 terminated with a matched load, and *M*_21_ is the measured transmission coefficient from Port 1 to Port 2 with Port 2 terminated with a matched load. Due to symmetry, we have *M*_22_ = *M*_11_ and *M*_12_ = *M*_21_, where *M*_22_ is the reflection coefficient at Port 2 with Port 1 terminated with a matched load, and *M*_12_ is the transmission coefficient from Port 2 to Port 1 with Port 1 terminated with a matched load. The proposed method is formulated using the scattering parameter instead of the transmission matrix, which is often employed in calibration approaches that use two or more LINE measurements, such as the TRL method or the LRL method [36,37]. In the proposed method, we use only one THRU measurement, and thus, the use of scattering parameters is more convenient.

There are three unknowns (*S*_11_, *S*_22_, and *S*_21_*S*_12_) to be determined, while there are only two equations (Equations (1) and (2)). An additional equation can be obtained from the REFLECT measurement shown in Figure 1b. From the REFLECT measurement, we obtain a reflection coefficient provided by

(3) $$Q_{11}=\frac{b_{1}}{a_{1}}=S_{11}+\frac{S_{21}S_{12}\Gamma }{1-\Gamma S_{22}}$$

We can manipulate Equations (1)–(3) to obtain the following system of linear equations for *S*_11_, *S*_22_, and ∆.

(4) $$S_{11}+M_{21}TS_{22}=M_{11}$$

(5) $$M_{11}TS_{22}-T\Delta =M_{21}$$

(6) $$S_{11}+\Gamma Q_{11}S_{22}-\Gamma \Delta =Q_{11}$$

where

(7) $$\Delta =S_{11}S_{22}-S_{12}S_{21}$$

Solving Equations (4)–(6) for *S*_22_ yields

(8) $$S_{22}=\frac{T(M_{11}-Q_{11})+\Gamma M_{21}}{T\left[\Gamma (M_{11}-Q_{11})+TM_{21}\right]}$$

The remaining two knowns, *S*_11_ and ∆, can be obtained using

(9) $$S_{11}=M_{11}-M_{21}TS_{22}$$

(10) $$\Delta =M_{21}/T-M_{11}S_{22}$$

From Equations (8)–(10), we obtain,

(11) $$S_{21}S_{12}=S_{11}S_{22}-\Delta$$

For reciprocal devices, the transmission coefficient can be obtained using the following equation.

(12) $$S_{21}=S_{12}=\pm \sqrt{S_{11}S_{22}}$$

The sign ambiguity in Equation (12) is resolved using rough knowledge of the electrical length of the device.

Calibration Equations (4)–(6) are singular when the determinant of a matrix–equation representation of a system of linear equations (Equations (4)–(6)) is zero. This is the same condition in which the denominator of Equation (8) is zero, viz.,

(13) $$T\;\Gamma (M_{11}-Q_{11})+M_{21}T^{2}=0$$

from which we obtain

(14)  T=0

or

(15) $$\;\Gamma (Q_{11}-M_{11})=M_{21}T$$

Equation (14) is trivial, and we can use Equation (15) to derive a singular condition. We can plug Equations (1)–(3) into Equation (15) to obtain

(16) $$\Gamma \left(S_{11}+\frac{S_{21}S_{12}\Gamma }{1-\Gamma S_{22}}-S_{11}-\frac{S_{21}S_{12}T^{2}S_{22}}{1-S_{22}^{2}T^{2}}\right)=\frac{S_{21}S_{12}T^{2}}{1-S_{22}^{2}T^{2}}$$

which is simplified to the following equation:

(17) $$\Gamma \;\left[\Gamma (1-S_{22}^{2}T^{2})-T^{2}S_{22}(1-\Gamma S_{22})\right]=T^{2}(1-\Gamma S_{22})$$

A further reduction of Equation (17) yields

(18) $$\Gamma (\Gamma -T^{2}S_{22})=T^{2}(1-\Gamma S_{22})$$

from which we obtain the following condition for the singularity.

(19) $$\Gamma^{2}=T^{2}\;\;\;\;\to \;\;\;\;\;\Gamma =T\;\;\mathrm{or}\;\Gamma =-T$$

Equation (19) is a general relation that applies to any calibration scheme that uses the LINE and REFLECT measurements for two identical devices. The LINE measurement and the REFLECT measurement are no longer independent of each other whenever the condition of Equation (19) is satisfied for arbitrary *T* and Γ, which is to be avoided for accurate measurements.

**Figure 2:**
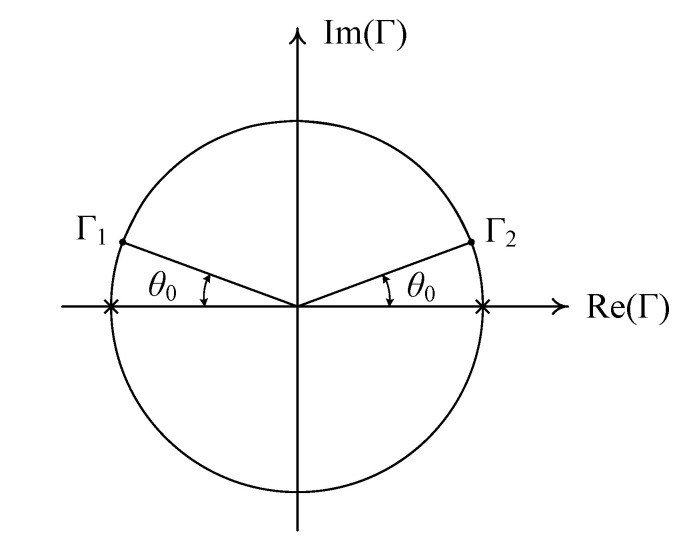
Singularities in scattering parameter extraction and the reflection coefficient of the offset SHORT with *T* =1.

Specifically, when *T* = 1 (a THRU configuration), an OPEN standard (Γ = 1) or a SHORT standard (Γ = −1) cannot be used in the THRU–REFLECT method. Instead, one can use an offset SHORT, which is easy to implement in hollow cylindrical transmission lines such as rectangular and circular waveguides. Two singular points, Γ = ±1, provided by Equation (19) are denoted by × in Figure 2 in the case of the THRU-connected back-to-back measurement (*T* = 1).

For measurement frequency from *f*_1_ to *f*_2_, the reflection coefficient of the REFLECT standard, Γ, is set to be equal to Γ_1_ and Γ_2_ at frequencies *f*_1_ and *f*_2_, respectively, as shown in Figure 2, where Γ_1_ and Γ_2_ are provided by

(20) $$\Gamma_{1}=-e^{-j\theta_{0}}=e^{j(\pi -\theta_{0})}\;\;\;(f=f_{1})$$

(21) $$\Gamma_{2}=-e^{-j(\pi -\theta_{0})}=e^{j\theta_{0}}\;\;\;\;\;\;\;(f=f_{2})$$

For accurate measurements, a minimum value of the angular distance, *θ*_0_, of the reflection coefficient of the REFLECT standard from the singularities of the THRU measurement ranges from 10° to 30° [38].

Let the waveguide length of the offset SHORT be *L*. Then, the input reflection coefficient of the offset SHORT is provided by

(22) $$\Gamma =-e^{-j\theta }=e^{j(\pi -\theta )}$$

where *θ* is the two-way phase shift in the waveguide of length *L*, which is provided by

(23) $$\theta =\frac{4\pi L}{\lambda_{g}}$$

where *λ_g_* is the wavelength in the waveguide provided by

(24) $$\lambda_{g}=\frac{c}{\sqrt{f^{2}-f_{c}^{2}}}$$

where *c* is the speed of light in the material that fills the waveguide, and *f_c_* is the cutoff frequency of the propagating mode in the waveguide. For the rectangular waveguide TE_10_ mode, *f_c_* is provided by *c*/(2*a*), where *a* is the width of the broad wall. It is important to note that only a single mode should propagate in the waveguide since all of the quantities we have mentioned (*S*_11_, *S*_21_, *S*_21_*S*_12_, *T*, and Γ) are defined for a single propagating mode, which is usually the lowest-order dominant mode.

The optimum length of the offset SHORT can now be obtained as follows. For *f*_1_ ≤ *f* ≤ *f*_2_, we require

(25) $$\theta_{0}\leq \theta \leq \pi -\theta_{0}$$

from which we obtain

(26) $$\theta_{0}=\frac{4\pi L}{\lambda_{g1}}\;\;\;\&\;\;\;\;\pi -\theta_{0}=\frac{4\pi L}{\lambda_{g2}}$$

Finally, from Equation (26), we obtain

(27) $$L=\frac{\lambda_{g1}\lambda_{g2}}{4(\lambda_{g1}+\lambda_{g2})}$$

In the next section, we will present an example of the application of the proposed method using the numerical simulation of a back-to-back configuration of two coaxial-to-waveguide adapters.

## 3. Example of Scattering Parameter Extraction

In this section, an example is presented for the THRU–REFLECT method of scattering parameter extraction based on the theory described in Section 2. *M*_11_ and *M*_21_ (Equations (1) and (2)) from the THRU measurement and *Q*_11_ (Equation (3)) from the REFLECT measurement are obtained by the numerical simulation of respective structures. Any numerical simulation has finite accuracy so that it can mimic actual measurements. The widely used CST Studio Suite^TM^ V. 2022 is used for the simulation.

**Figure 3:**
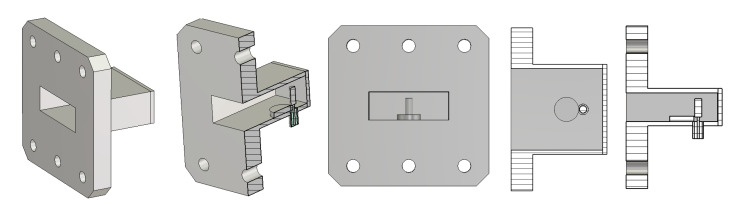
Coaxial-to-reduced-height rectangular waveguide transition as an example of scattering parameter extraction from back-to-back measurements. Various views are presented to aid in the understanding of the transition.

To show the workings of the proposed method, we use a coaxial-to-reduced-height rectangular waveguide transition, shown in Figure 3. The transition consists of a coaxial probe and a tuning post, all of which are positioned in the waveguide center line. With centered symmetric structures and the excitation of the even-symmetric field of the TE_10_ mode, the TE_20_ mode with its odd-symmetric field is not excited. The transition is of a very wideband design, which is made possible by reducing the waveguide height, *b*, so that the cutoff frequencies of the TE_11_ and TE_30_ modes coincide with each other, which is satisfied when *b* = *a*/√8 = 0.354*a* [39], where *a* and *b* are the width of the broad and narrow walls of the rectangular waveguide. Therefore, the transition can operate from the cutoff frequency of the dominant TE_10_ mode to that of the TE_11_ and TE_30_ modes. We use the broad wall width *a* = 19.05 mm (the WR-75 standard waveguide) and the narrow wall height *b* = 6.74 mm in our example so that the transition can operate from 7.87 GHz to 23.62 GHz. The transition has been designed so that its bandwidth is as large as possible.

**Figure 4:**
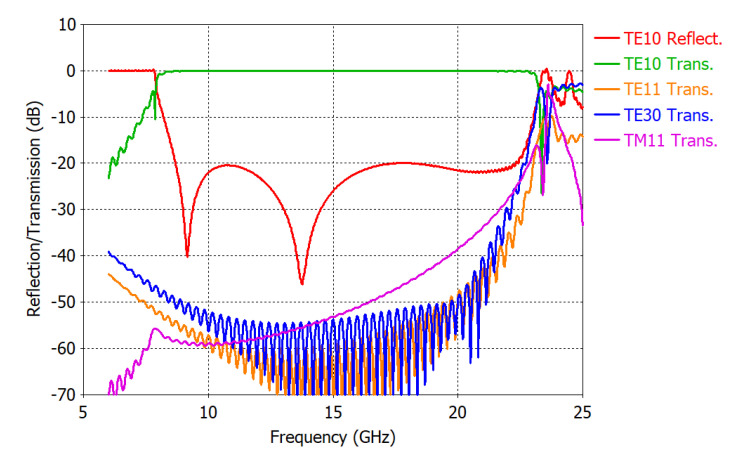
Reflection and transmission coefficients of the coaxial-to-waveguide transition.

Figure 4 shows the simulated TE_10_ mode reflection coefficient and higher-order mode transmission coefficients of the transition. The reflection coefficient corresponds to *S*_11_ of the transition, which needs to be extracted by the THRU–REFLECT method. In Figure 4, we can observe that the transition’s reflection coefficient is less than −20 dB at 8.65–22.31 GHz. Above 22.31 GHz, the reflection coefficient of the TE_10_ mode and the transmission coefficients of higher-order TE_11_, TE_30_, and TM_11_ modes increase rapidly.

**Figure 5:**
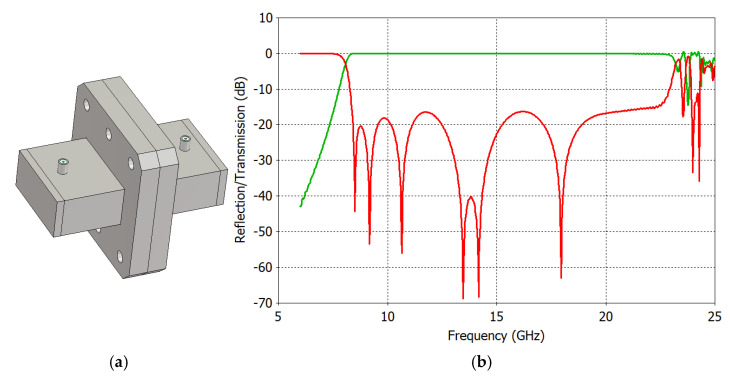
(**a**) Two identical coaxial-to-waveguide transitions in a back-to-back configuration and (**b**) their reflection (in red) and transmission (in green) coefficients.

Figure 5a shows two identical transitions joined in a back-to-back configuration and Figure 5b shows their TE_10_ mode reflection and transmission coefficients, which correspond to the magnitudes of *M*_11_ and *M*_21_ provided by Equations (1) and (2), respectively. In order to extract the scattering parameters (*S*_11_, *S*_22_, and *S*_21_) of the transition, we need the phase information as well, and it will be determined when we compare the extracted scattering parameters with the simulated ones.

Comparing Figure 4 with Figure 5b, we note that the reflection coefficient of the transition in a back-to-back configuration is significantly different from that of a single transition and that the back-to-back measured values can hardly represent the performance of the designed transition shown in Figure 4. Due to multiple reflections between two transitions, more local maxima and minima appear in the reflection coefficient curve. The maximum reflection coefficient is increased from −20 dB to −16.3 dB in the operating frequency range of the transition.

**Figure 6:**
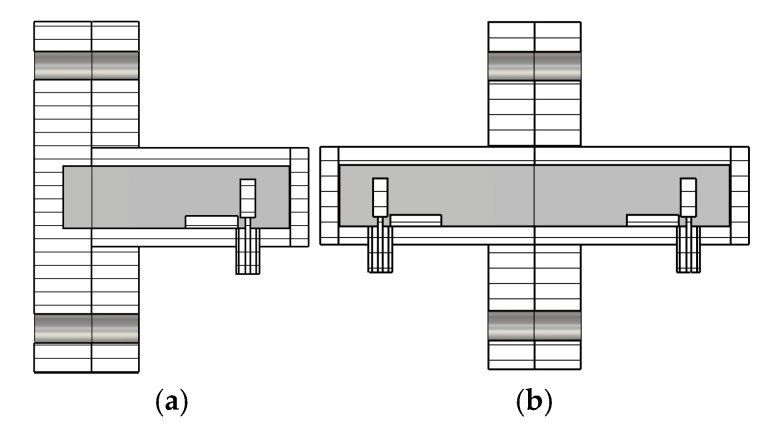
Configurations for (**a**) an offset SHORT measurement and (**b**) a THRU or back-to-back measurement.

Figure 6 shows the configurations for the offset SHORT and the THRU or back-to-back measurements. Figure 6a is the structure for a measurement with an offset SHORT, while Figure 6b shows a configuration for a THRU measurement. First, we make a measurement with an offset SHORT through numerical simulation using CST Studio Suite^TM^ V. 2022. The length of the offset SHORT is determined to be 3.10 mm for a frequency range of 8–24 GHz according to Equation (27). With an offset SHORT length of 3.10 mm, the two-way phase delay is 10.5° at 8 GHz and 168.8° at 24 GHz. The reflection coefficient, *Q*_11_, of the offset SHORT measurement is obtained through the numerical simulation of the structure of Figure 6a. Simulation is performed at 1001 frequency points from 6 GHz to 25 GHz.

Next, we make a measurement of the transition with a THRU connection. The reflection coefficient, *M*_11_, and the transmission coefficient, *M*_21_, are simulated using the back-to-back configuration of Figure 6b. The values of *M*_11_ and *M*_21_ are obtained at 1001 frequency points from 6 GHz to 25 GHz. A code has been written to read the numerical data of the magnitudes and phases of *M*_11_, *M*_21_, and *Q*_11_ obtained by the simulation and to extract the scattering parameters of a single transition using the equations presented in Section 2.

**Figure 7:**
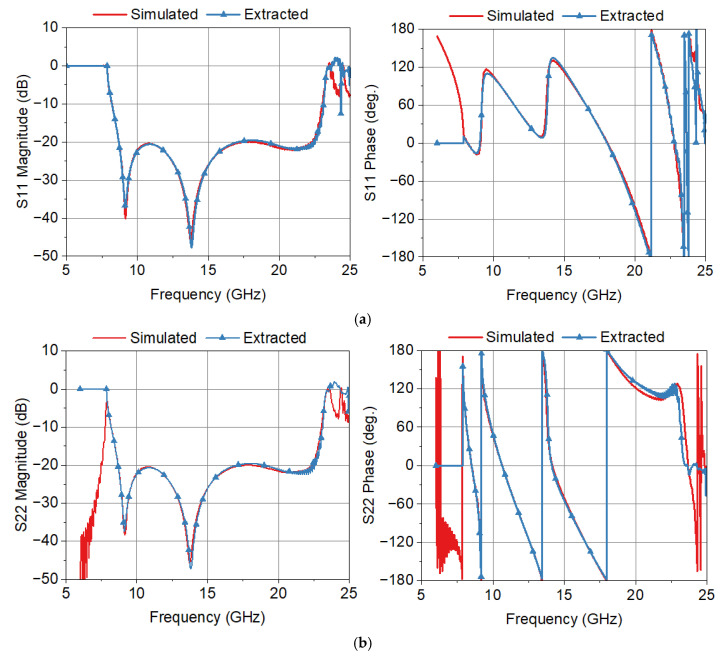

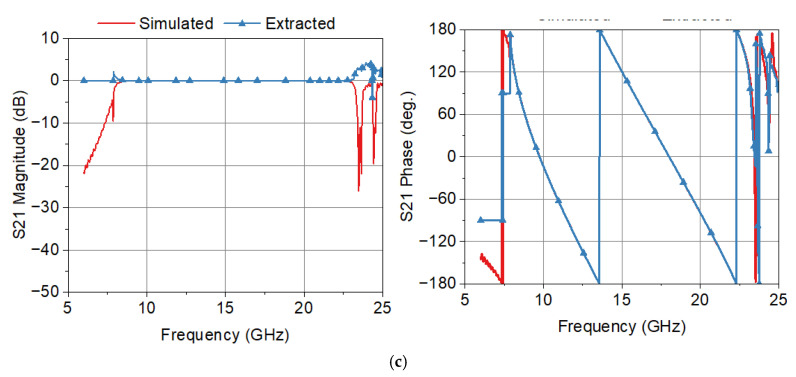
Comparison of the simulated and extracted scattering parameters of the transition. (**a**) *S*_11_, (**b**) *S*_22_, and (**c**) *S*_21_.

Figure 7 shows a comparison of the simulated and extracted scattering parameters of a single transition. Data for frequencies less than the cutoff frequency (7.87 GHz) of the waveguide are not valid and are used only for graphing purposes. We note in Figure 7 that the agreements between the simulated and extracted scattering parameters are excellent. As the frequency approaches the cutoff frequency of the TE_11_ and TE_30_ modes, there are some noticeable errors in the extracted scattering parameters. This can be attributed to the increased levels of higher-order modes generated in the transition.

The proposed method yields accurate results at frequencies where only the dominant mode can propagate. In order to include the effects of the higher-order modes and, thus, to increase the frequency range of device characterization, an advanced method proposed in [40] can be used, where the same calibration standards as the ones used in this paper are employed, but a sophisticated nontermination algorithm is used that employs the generalized scattering matrix (GSM) and the genetic algorithm–gradient descent (GA-GD) method-based optimization technique.

For a device that employs higher modes and a dominant mode, structures for extracting specific modes are prepared, and measurements are made for a combination of modes, as well as for specific modes. To extract the device characteristics, the generalized scattering matrix is used, which includes interactions between all of the modes present in the device [41].

Table 1 shows numerical values used for the *S*-parameter extraction at 10, 15, and 20 GHz, along with the extracted scattering parameters of a single transition. The difference between the actual and extracted magnitudes of *S*_11_ and *S*_22_ is less than 0.6 dB. The maximum phase difference in the phases of *S*_11_ and *S*_22_ is less than 10°. Errors in the phases of *S*_11_ and of *S*_22_ are increased when the magnitudes of *S*_11_ and *S*_22_ are small, for example, less than −30 dB. Agreements in the magnitude and phase of *S*_21_ are excellent. This is due to the fact that the magnitude of *S*_21_ is close to 0 dB.

Table 2 shows error statistics for the scattering parameter extraction. The difference represents the extracted value minus the simulated value. The standard deviation is less than 0.7 dB in the magnitudes of *S*_11_ and *S*_22_, while it is less than 4.5° in the phases of *S*_11_ and *S*_22_. Large phase errors in *S*_11_ and *S*_22_ at 9.12 GHz and at 13.77 GHz are due to the smallness of the magnitude of *S*_11_ and *S*_22_. Agreement in *S*_21_ is excellent, with the standard deviation of the magnitude and phase of *S*_21_ being 0.18 dB and 0.077°, respectively. Error in *S*_21_ is increased as the frequency approaches the cutoff frequency (7.87 GHz).

Figure 7 and Table 1 and Table 2 show that the proposed THRU–REFLECT method offers an accurate approach to extracting the scattering parameters of a single device from a back-to-back measurement of waveguide components for a 3:1 bandwidth with one offset SHORT measurement and one THRU measurement. With a simple modification, the proposed method can be applied to the back-to-back measurements of other types of microwave devices. For example, the proposed method can be applied to microstrip devices using an offset SHORT or OPEN standard for the REFLECT measurement.

In Table 3, the proposed method is compared with other methods applicable to the characterization of back-to-back connected devices. The THRU–LINE method [42] and the THRU–MATCH method [43] are more complicated than the proposed method. The former requires four flange connections, while the latter uses a matched termination, which is costly to realize. The THRU-Only method [44] is simpler than the proposed method since it requires only a THRU connection or a back-to-back connection and one two-port measurement. However, its applicability is limited since the condition *S*_11_ = *S*_22_ is assumed. The 2x-THRU method [45] is widely used and implemented in high-end vector network analyzers (VNAs), referred to as AFR (Automatic Fixture Removal). It uses a LINE standard that is long enough for the time–domain separation of a short impulse signal. It requires time–domain gating software functionality, as well as a broadband (e.g., from 10 MHz to 50 GHz) VNA. Thus, the 2x-THRU method is useful only for device measurements using high-end VNAs.

The advantage of the proposed method lies in the fact that it requires a simple offset short, which is easy to implement in a waveguide, and that only three flange connections are made during measurements. The algorithm of the proposed method is simple, and it can be compactly implemented in low-end VNAs.

## 4. Conclusions

A full and comprehensive treatment has been presented for the THRU–REFLECT method for scattering parameter extraction from a back-to-back measurement of waveguide components. It has been shown that the scattering parameters of a single device can conveniently be obtained from a back-to-back measurement of two identical waveguide devices using the THRU–REFLECT method, which includes a reflection coefficient measurement with an offset SHORT before or after a THRU measurement. The singularity condition has been derived for a general LINE–REFLECT measurement. The optimum length of an offset SHORT or a reflecting load has been provided in terms of the guided wavelength at the start and end frequencies of the measurement. The workings of the proposed method have been shown by the numerical simulation of a broadband coaxial-to-reduced waveguide transition. It has been shown that with an offset SHORT of optimum length, the scattering parameters of a single device can be accurately extracted from back-to-back measurements over a 3:1 frequency range in the case of a reduced-height rectangular waveguide. Using the proposed method, the general practice in the microwave community of validating new waveguide devices with back-to-back measurements can now be upgraded to include the extraction of the scattering parameters of a single device in a simple and economical way. Areas of additional research include the analysis of measurement uncertainty due to the imperfect mating of waveguides and the application of the proposed method to the back-to-back measurements of other types of microwave devices.

## Figures and Tables

**Table 1:** Input data for and results of the *S*-parameter extraction.

Quantities (Units: dB, deg.)	Frequency (GHz)		
	**10**	**15**	**20**
Mag./Phase of *M*_11_	−18.86/61.84	−21.84/150.14	−17.35/−68.80
Mag./Phase of *M*_21_	−0.0561/−28.08	−0.0293/−119.67	−0.0837−159.54
Mag./Phase of *Q*_11_	−0.0002/105.18	−0.0000/−31.70	0.0019/−114.84
Mag./Phase of *T*	0/0	0/0	0/0
Mag./Phase of Γ	0/133.95	0/84.87	0/43.08
Mag./Phase of *S*_11_, Simulated	−22.05/105.16	−26.00/118.83	−21.60/−100.42
Mag./Phase of *S*_11_, Extracted	−22.37/102.98	−26.09/115.10	−21.02/−107.66
Mag./Phase of *S*_22_, Simulated	−22.04/46.00	−26.02/−51.19	−21.54/121.47
Mag./Phase of *S*_22_, Extracted	−22.36/48.53	−26.10/−54.91	−21.15/130.76
Mag./Phase of *S*_21_, Simulated	−0.0402/−14.17	−0.0247/120.23	−0.0428/−79.69
Mag./Phase of *S*_21_, Extracted	0.0255/−14.21	0.0103/120.23	0.0308/−79.53

**Table 2:** Error statistics in the *S*-parameter extraction.

Differences	Average	Standard Deviation	Max./Freq. (GHz)	Min./Freq. (GHz)
In Mag. of *S*_11_ (dB)	0.0088	0.63	3.28/9.17	−3.50/13.85
In Phase of *S*_11_ (deg.)	−1.77	4.34	12.32/9.14	−31.16/13.77
In Mag. of *S*_22_ (dB)	0.0098	0.69	2.38/13.66	−4.19/13.90
In Phase of *S*_22_ (deg.)	2.18	4.34	38.03/13.77	−25.56/9.12
In Mag. of *S*_21_ (dB)	0.0093	0.18	2.19/8.01	0.0011/9.06
In Phase of *S*_21_ (deg.)	0.040	0.077	0.57/8.09	−0.61/8.033

**Table 3:** Comparison of the methods applicable to device characterization in a back-to-back configuration.

Methods	Number of Interface Connections	Number of Calibration Standards	Applicable to Asymmetric Devices?	Features
THRU-LINE [42]	4	1	No	Two 2-port measurements. One LINE standard required. Complexity: medium.
THRU-MATCH [43]	3	1	No	One 2-port measurement and one 1-port measurement. MATCH standard is costly. Complexity: high.
THRU-Only [44]	2	0	No	One 2-port measurement. Assumption: *S*_11_ = *S*_22_ (Limited applications). Complexity: low.
2x-THRU [45]	2	1	Yes	Time–domain gating used. One long LINE standard used. Complexity: hardware = low; software = high
Proposed Method	3	1	No	One 1-port measurement and one 2-port measurement. Offset SHORT standard required. Complexity: low.

## Data Availability

The data presented in this study are available in this article.
